# Analysis of Contraceptive Use Among Immigrant Women Following Expansion of Medicaid Coverage for Postpartum Care

**DOI:** 10.1001/jamanetworkopen.2021.38983

**Published:** 2021-12-15

**Authors:** Maria I. Rodriguez, Megan Skye, Stephan Lindner, Aaron B. Caughey, Ana Lopez-DeFede, Blair G. Darney, K. John McConnell

**Affiliations:** 1 Department of Obstetrics and Gynecology, Oregon Health & Science University, Portland; 2 Center for Health Systems Effectiveness, Oregon Health & Science University, Portland; 3 Institute for Families in Society, University of South Carolina, Columbia; 4 Divisionof Complex Family Planning, Department of Obstetrics and Gynecology, Oregon Health & Science University, Portland

## Abstract

**Importance:**

Access to postpartum care is restricted for low-income women who are recent or undocumented immigrants enrolled in Emergency Medicaid.

**Objective:**

To examine the association of a policy extending postpartum coverage to Emergency Medicaid recipients with attendance at postpartum visits and use of postpartum contraception.

**Design, Setting, and Participants:**

This cohort study linked Medicaid claims and birth certificate data from 2010 to 2019 to examine changes in postpartum care coverage on postpartum care and contraception use. A difference-in-difference design was used to compare the rollout of postpartum coverage in Oregon with a comparison state, South Carolina, which did not cover postpartum care. The study used 2 distinct assumptions to conduct the analyses: first, preintervention differences in postpartum visit attendance and contraceptive use would have remained constant if the policy expanding coverage had not been passed (parallel trends assumption), and second, differences in preintervention trends would have continued without the policy change (differential trend assumption). Data analysis was performed from September 2020 to October 2021.

**Exposures:**

Medicaid coverage of postpartum care.

**Main Outcomes and Measures:**

Attendance at postpartum visits and postpartum contraceptive use, defined as receipt of any contraceptive method within 60 days of delivery.

**Results:**

The study population consisted of 27 667 live births among 23 971 women (mean [SD] age, 29.4 [6.0] years) enrolled in Emergency Medicaid. The majority of all births were to multiparous women (21 289 women [76.9%]; standardized mean difference [SMD] = 0.08) and were delivered vaginally (20 042 births [72.4%]; SMD = 0.03) and at term (25 502 births [92.2%]; SMD = 0.01). Following Oregon’s expansion of postpartum coverage to women in Emergency Medicaid, there was a large and significant increase in postpartum care visits and contraceptive use. Assuming parallel trends, postpartum care attendance increased by 40.6 percentage points (95% CI, 34.1-47.1 percentage points; *P* < .001) following the policy change. Under the differential trends assumption, postpartum visits increased by 47.9 percentage points (95% CI, 41.3-54.6 percentage points; *P* < .001). Postpartum contraception use increased similarly. Under the parallel trends assumption, postpartum contraception within 60 days increased by 33.2 percentage points (95% CI, 31.1-35.4 percentage points; *P* < .001). Assuming differential trends, postpartum contraception increased by 28.2 percentage points (95% CI, 25.8-30.6 percentage points; *P* < .001).

**Conclusions and Relevance:**

These findings suggest that expanding Emergency Medicaid benefits to include postpartum care is associated with significant improvements in receipt of postpartum care and contraceptive use.

## Key Points

**Question:**

Is the inclusion of postpartum care as a benefit in Emergency Medicaid, a program of restricted Medicaid services for low-income and pregnant recent immigrants, associated with an improvement in postpartum care attendance and contraception use?

**Findings:**

In this cohort study of 27 667 live births among 23 971 women, the inclusion of postpartum care benefits for women enrolled in Emergency Medicaid was associated with increased attendance at postpartum visits and increased use of all forms of effective contraception.

**Meaning:**

These findings suggest that when postpartum care is covered, women enrolled in Emergency Medicaid use the expanded services, including contraception.

## Teaser

This cohort study examines whether a policy extending Emergency Medicaid postpartum coverage to low-income women who are recent or undocumented immigrants is associated with increased attendance at postpartum visits and use of postpartum contraception.

## Introduction

The postpartum period is critical for maternal and newborn health in preventing and treating acute complications as well as establishing the foundation for long-term health.^1^ Comprehensive postpartum care includes a full assessment of physical, psychological, and social well-being. Its components include counseling on the importance of birth spacing and providing the contraceptive method of their choice.^1^ An absence of postpartum care has been associated with unintended pregnancy, short interpregnancy intervals, exacerbation of chronic diseases, and preterm birth.^2,3,4,5^

Low-income immigrant women represent a uniquely vulnerable group who are systematically excluded from postpartum care.^6^ By federal law, undocumented immigrants and documented immigrants who have been in the US for less than 5 years are not eligible for full-benefit or Traditional Medicaid.^6^ Instead, coverage for these women is limited to Emergency Medicaid, which restricts benefits to life-threatening conditions, including hospital admission for childbirth. No prenatal or postpartum care, including contraception, is covered.^6^ Nationally, it is estimated that there are 3.7 million unauthorized immigrant women who are of reproductive age living in the US, with approximately 7.5% giving birth annually.^7,8^

Federal policy changes to the Children’s Health Insurance Program, enacted in 2002 and renewed in 2009, gave states new options for Emergency Medicaid recipients to provide prenatal care coverage, regardless of their legal status or date of entry to the US.^9,10^ Oregon is 1 of 19 states that expanded prenatal care—but not postpartum care—to the Emergency Medicaid population using these policy options.^9^ The expansion of prenatal care significantly improved the receipt of recommended screening tests and vaccinations.^11^ However, the use of postpartum contraception did not change, indicating that policies that focused strictly on expanding prenatal care for Emergency Medicaid might not be sufficiently comprehensive for these new mothers.

In 2017, Oregon passed The Reproductive Health Equity Act, which included coverage for 60 days of postpartum care, including contraception, for the Emergency Medicaid population.^2^ The act went into effect on April 1, 2018. This study assessed the impact of this policy on the Emergency Medicaid population, leveraging 8 years of linked birth certificate and Medicaid claims data. We compared Oregon with South Carolina, which did not expand coverage beyond the federal minimum. We included Oregon and South Carolina because both states have experienced similar growth in their immigrant population and have comparable immigrant populations, in terms of size and country of origin, residing in each state. Our primary focus was on the association of the policy change with subsequent attendance at a postpartum visit and receipt of postpartum contraception within 60 days of delivery.

## Methods

### Data

**Figure 1.**
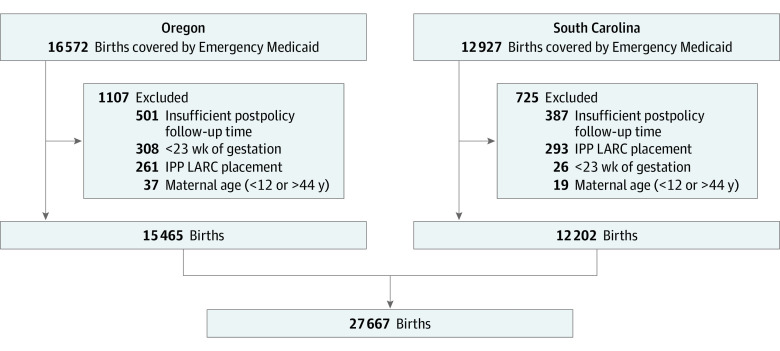
Study Cohort Creation Diagram, Oregon and South Carolina, 2014-2019 IPP indicates immediate postpartum; LARC, long-acting reversible contraception.

We used linked Medicaid claims and birth certificate data from Oregon and South Carolina to conduct a retrospective cohort study (Figure 1). Our study period was January 1, 2014, to December 31, 2019, to allow for 40 months of data before the policy change and 18 months after the policy change. We restricted our study sample to births occurring between January 1, 2014, to October 31, 2019, to allow a minimum of 60 days of postpartum follow-up. We followed the Strengthening the Reporting of Observational Studies in Epidemiology (STROBE) reporting guideline.^12^ The institutional review board at Oregon Health & Science University approved the study. The data are publicly available and deidentified, so informed consent was not obtained, in accordance with 45 CFR §46.

We restricted our study population to births among Emergency Medicaid recipients during our study period (Figure 1). A prerequisite for enrollment in Emergency Medicaid is being both low-income and a noncitizen. We therefore used Emergency Medicaid as a measure of immigrant status. We included women aged 15 to 44 years in our study sample. We excluded births that were less than 23 weeks or greater than 44 weeks gestation. To isolate associations with the policy change, which focused on expanding postpartum care after discharge, we excluded women who had long-acting reversible contraception (LARC), such as intrauterine devices and implants, placed immediately postpartum. Both states had philanthropic grants or research studies enabling access to immediate postpartum LARC outside of the policy change. Therefore, we excluded women using these methods to focus on the association of the policy change with postpartum contraceptive use.^13^

Our outcomes were attendance at postpartum visits and the receipt of any postpartum contraception method within 60 days. Our independent variable was a binary variable indicating the expansion of postpartum coverage to the Emergency Medicaid population. During our study period, South Carolina did not cover prenatal or postpartum care for Emergency Medicaid recipients. Oregon covered prenatal care and began providing postpartum care coverage statewide for the Emergency Medicaid population on April 1, 2018.^2^ All contraceptive methods approved by the Food and Drug Administration were covered. We identified births and date of delivery using a previously published algorithm.^14^ We identified postpartum visits through diagnosis codes indicative of postpartum care or preventative gynecological care (see eTable 1 in the Supplement). We calculated the timing of the postpartum visit as the difference in days between the date of delivery and the date of the first postpartum visit claim. Women with a postpartum visit claim within 60 days of delivery were classified as having a postpartum visit. We identified postpartum contraception through procedure and National Drug Codes associated with receipt of contraception (see eTable 2 in the Supplement). We calculated the timing of contraception as the number of days between the date of delivery and the date of the contraception claim. Women with a claim for postpartum contraception within 60 days of delivery were classified as receiving postpartum contraception.

Secondary outcomes focused on how the policy change was associated with receipt of different types of moderately or highly effective contraception: sterilization, LARC, and short-acting hormonal methods (eg, injectable progestin, oral contraception, patch, and ring).^15^ We identified sterilization, LARC, and injectable progestin through inpatient or outpatient Medicaid procedure claims. We identified other short-acting methods using National Drug Codes through Medicaid pharmacy claims for oral contraception, patch, or ring.

### Variables

We abstracted demographic and clinical information from the birth certificate files and claims data. We included the demographic variables of maternal age (<20, 20-34, and ≥35 years), multiparity, race and ethnicity, county of residence (metropolitan, nonmetropolitan, missing), and state. We included race and ethnicity in this study because of previous work demonstrating significant differences by race and ethnicity, by payor type, and by postpartum contraceptive use. We included the clinical variables of adequate prenatal care (≥7 prenatal visits), mode of delivery, preterm birth (births <37 weeks gestation), and pregnancy complications (preexisting diabetes, preexisting hypertension, gestational diabetes, and gestational hypertensive disorders).

### Statistical Analysis

To compare the distribution of baseline covariates between Oregon and South Carolina, we calculated absolute standardized mean differences (SMDs) for demographic and clinical covariates between states. The SMD is not affected by sample size and is thus useful for making comparisons in large observational studies. We considered an absolute SMD of less than 0.1 to denote minor differences between groups.^16^

To compare changes in our outcomes in Oregon vs South Carolina from the prepolicy period to the postpolicy period, we used a difference-in-difference (DID) design. DID analyses relied on the assumption that the composition of the 2 states were stable over time; traditional DID analyses also assumed that the time trends in outcomes were the same in the 2 states in the prepolicy period (ie, parallel pretrends). However, if the prepolicy trends differed between Oregon and South Carolina, traditional DID might result in biases estimations.^17^ To mitigate this potential source of bias, we also conducted a second DID analysis where we assumed that differences observed in the prepolicy period between Oregon and South Carolina would have continued to change at the same rate in the absence of the policy change. This second analysis provides an estimate of changes in Oregon, compared with our South Carolina comparison group, or the net of changes that would be expected from the trends observed before the policy change.^18^

We selected covariates for models on the basis of clinical or reported associations with our outcomes.^5,19^ In our models for all of our outcomes, we adjusted for maternal age, rural location, cesarean delivery, preterm birth, and pregnancy complications. SEs were clustered at the county level.

We observed differences in how race and ethnicity were coded in the 2 states, with a large proportion of women in South Carolina being coded as having other or unknown race or ethnicity. We therefore conducted sensitivity analyses comparing baseline covariates between those with an other or unknown race or ethnicity to those with all other races and ethnicities (eTable 3 in the Supplement). Nationally, the majority of Emergency Medicaid recipients are Latina and female. Latina women also experience significant disparities in contraceptive use.^10,11,12^ We therefore conducted one set of subanalyses that restricted the study population to Latina women (eTable 4 in the Supplement). Results were robust, and we present results for the whole population only.

For all of our DID models, we conducted 2-sided χ^2^ tests with an α level of .05, and for our secondary outcomes, used a Bonferroni correction to account for multiple comparisons. We used R statistical software version 4.0.3 (R Project for Statistical Computing) to conduct our analyses. Data analysis was performed from September 2020 to October 2021.

## Results

Our study sample included 27 667 live births among 23 971 women (mean [SD] age, 29.4 [6.0] years) (Table 1). More births in our sample occurred in the Oregon cohort than the South Carolina comparison group (15 465 births [55.9%] vs 12 202 births [44.1%]). The majority of all births were to multiparous women (21 289 women [76.9%]; SMD = 0.08) and were delivered vaginally (20 042 births [72.4%]; SMD = 0.03) and at term (25 502 births [92.2%]; SMD = 0.01). Women giving birth in Oregon were more likely than those in South Carolina to be Latina (12 798 women [82.8%] vs 5610 women [46.0%]; SMD = 1.27), aged 35 years or older (3749 women [24.2%] vs 2297 women [18.8%]; SMD = 0.17), and living in metropolitan counties (12 964 women [83.8%] vs 7543 women [61.8%]; SMD = 0.64).

**Table 1.** Demographic and Clinical Characteristics of Births Among Emergency Medicaid Recipients in Oregon and South Carolina, 2014-2019

Characteristic	Participants, No. (%) (N = 27 667)^a^		Standardized mean difference
	Oregon (n = 15 465)	South Carolina (n = 12 202)	
Maternal age at birth, y			
<20	483 (3.1)	689 (5.6)	0.17
20-34	11 233 (72.6)	9216 (75.5)	
≥35	3749 (24.2)	2297 (18.8)	
Multiparous	12 119 (78.4)	9170 (75.2)	0.08
Race or ethnicity			
American Indian or Alaska Native	2 (0.0)	68 (0.6)	1.10
Asian^b^	995 (6.4)	372 (3.0)	
Black	306 (2.0)	167 (1.4)	
Latina	12 798 (82.8)	5610 (46.0)	
Native Hawaiian or Pacific Islander^c^	519 (3.4)	66 (0.5)	
White	675 (4.4)	330 (2.7)	
Other or unknown^d^	170 (1.1)	5589 (45.8)	
County of residence			
Metropolitan	12 964 (83.8)	7543 (61.8)	0.62
Nonmetropolitan	2221 (14.4)	2322 (19.0)	
Missing	280 (1.8)	2337 (19.2)	
Adequate prenatal care	13 619 (89.9)	9246 (76.0)	0.38
Pregnancy complications	3346 (21.6)	1781 (14.6)	0.18
Preterm birth	1191 (7.7)	974 (8.0)	0.01
Cesarean delivery	4341 (28.1)	3283 (26.9)	0.03

^a^ Individual variable denominators differ depending on missingness.

^b^ Category includes the variable reported subgroups Asian Indian, Chinese, Filipino, Japanese, Korean, Vietnamese, and other Asian.

^c^ Category includes the variable reported subgroups Native Hawaiian, Guamanian or Chamorro, Samoan, and other Pacific Islander.

^d^ Category includes the variable reported subgroups other and unknown.

Our standard DID models relied on the assumption of parallel trends. However, adjusted preintervention trends in Oregon differed appreciably from those in South Carolina in all of our models. Thus, we present estimates of our standard DID model and a model adjusting for preintervention trends.

**Figure 2.**
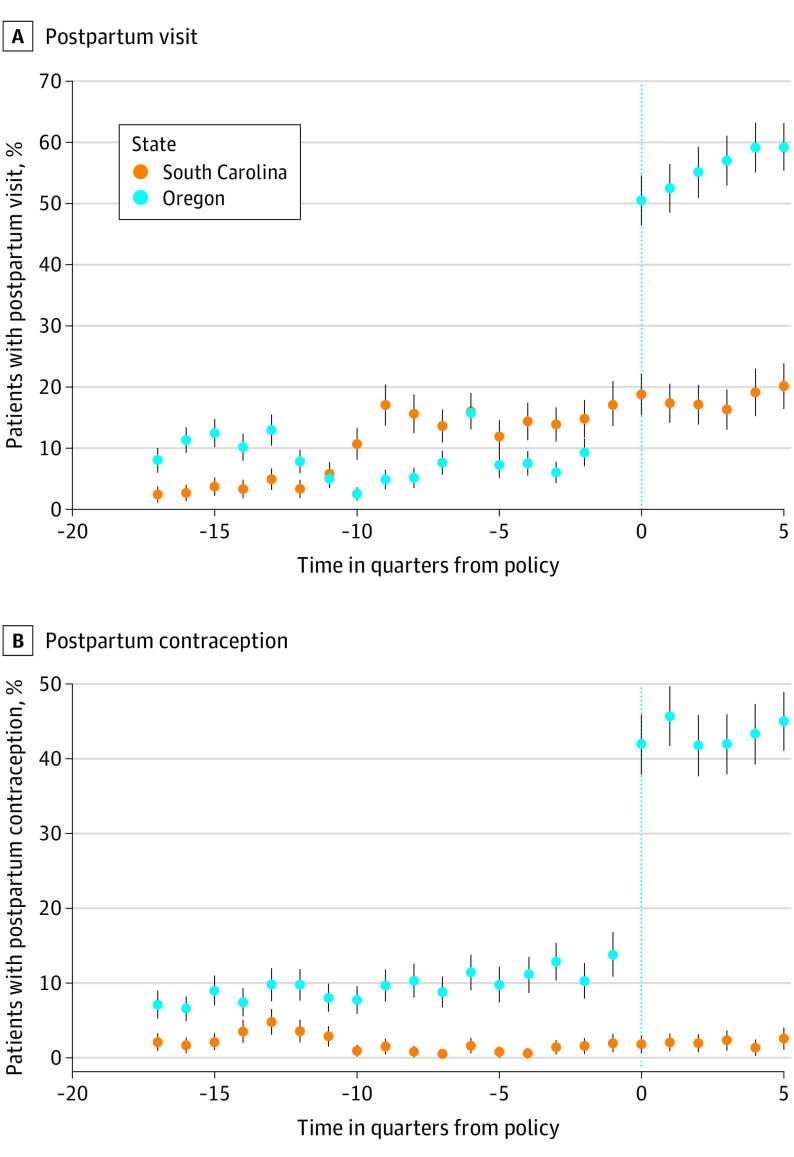
Unadjusted Trend Estimates of Postpartum Visit Attendance and Receipt of Postpartum Contraception Within 60 Days Among Emergency Medicaid Recipients in Oregon and South Carolina, 2014-2019 Graphs show data for attendance at a postpartum visit (A) and receipt of postpartum contraception (B) for 27 667 women. Dots denote mean estimates, and error bars denote 95% CIs.

Before the policy change, 8.8% of Emergency Medicaid enrollees (1050 women) attended a postpartum visit. After the policy, 55.6% of Emergency Medicaid enrollees (1933 women) attended a postpartum visit. In our adjusted DID model, assuming parallel trends, the policy was associated with an increase in postpartum visit attendance of 40.6 percentage points (95% CI, 34.1-47.1 percentage points; *P* < .001). Assuming differential trends, the policy was associated with an increase in postpartum visit attendance of 47.9 percentage points (95% CI, 41.3-54.6 percentage points; *P* < .001) (Table 2 and Figure 2; eTable 5 in the Supplement includes unadjusted models).

**Table 2.** Differential Changes From the Prepolicy Periods Through 2019, Oregon vs South Carolina, 2014-2019

Measure	Births in Oregon, No. (%) (N = 15 465)		Difference in prepolicy trends, Oregon vs South Carolina, % (95% CI)^a^	Difference-in-difference estimate (95% CI)^b^	
	Prepolicy (n = 11 988)	Postpolicy (n = 3477)		Assuming parallel trends^c^	Assuming differential trends^d^
Postpartum visit within 60 d	1050 (8.8)	1933 (55.6)	−0.71 (−1.16 to −0.25)	40.6 (34.1 to 47.1)	47.9 (41.3 to 54.6)
Postpartum contraception within 60 d	1129 (9.4)	1506 (43.3)	0.48 (0.29 to 0.68)	33.2 (31.1 to 35.4)	28.2 (25.8 to 30.6)
Tier 1					
Sterilization	921 (7.7)	525 (15.1)	0.25 (0.04 to 0.46)	6.8 (4.8 to 8.8)	4.1 (2.0 to 6.3)
Interval long-acting reversible contraception	63 (0.5)	683 (19.6)	0.09 (0.04 to 0.15)	18.7 (16.4 to 20.9)	17.7 (15.6 to 19.8)
Tier 2, hormonal methods	145 (1.2)	298 (8.6)	0.13 (0.07 to 0.20)	7.8 (5.8 to 9.7)	6.4 (4.2 to 8.5)

^a^ Prepolicy trend difference was estimated from an interaction between a linear prepolicy time trend and Oregon residence, adjusted for maternal age, nonmetropolitan location, cesarean delivery, and preterm gestational age. SEs clustered at the county level.

^b^ Difference-in-difference estimates were adjusted for maternal age, nonmetropolitan location, cesarean delivery, and preterm gestational age. SEs clustered at the county level.

^c^ Estimates assume that differences between Oregon and South Carolina would have remained constant had the policy not been implemented in Oregon.

^d^ Estimates assume that the prepolicy differences between Oregon and South Carolina would have continued had the policy not been implemented in Oregon.

We observed a similar increase in postpartum contraception use. Before the policy, 9.4% of Emergency Medicaid recipients (1129 women) received a contraceptive method by 60 days postpartum. After the policy, 43.3% of Emergency Medicaid enrollees (1506 women) received contraception within 60 days postpartum. Assuming parallel trends, the policy was associated with an increase in overall postpartum contraception use of 33.2 percentage points (95% CI, 31.1-35.4 percentage points; *P* < .001). Assuming differential trends, the policy was associated with an increase in overall postpartum contraception use of 28.2 percentage points (95% CI, 25.8-30.6 percentage points; *P* < .001), under the differential trends assumption (Table 2).

**Figure 3.**
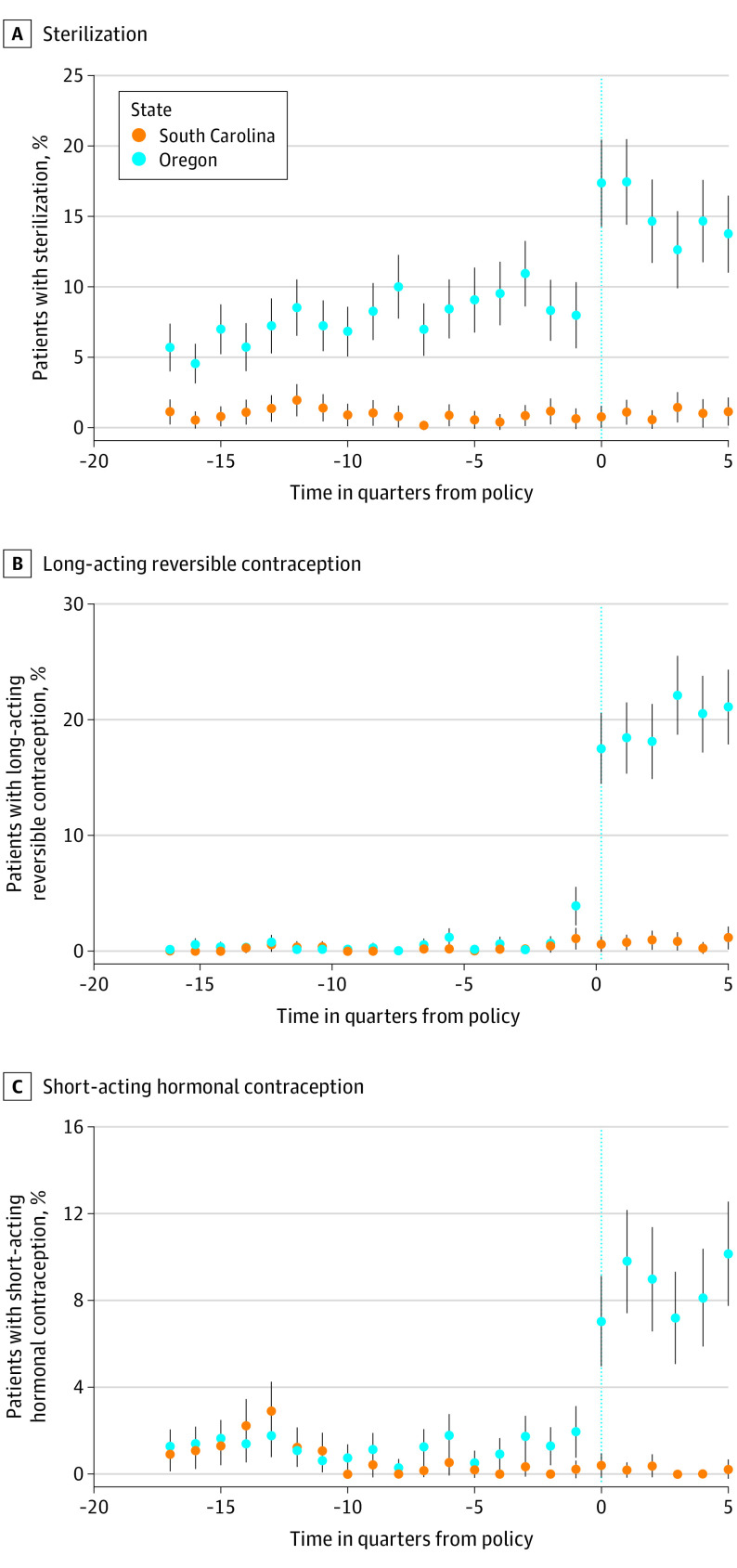
Unadjusted Trend Estimates of Receipt of Postpartum Contraception Within 60 Days by Contraception Type Among Emergency Medicaid Recipients in Oregon and South Carolina, 2014-2019 Graphs show data for sterilization (A), long-acting reversible contraception (B), and short-acting hormonal contraception (C) for 27 667 women. Dots denote mean estimates, and error bars denote 95% CIs.

We then examined changes in types of contraceptive methods. Before the policy change, sterilization was the most commonly used method of contraception, with 7.7% of Emergency Medicaid recipients (921 women) undergoing tubal ligation postpartum. The policy change was associated with an increase in sterilization procedures by 6.8 percentage points (95% CI, 4.8-8.8 percentage points; *P* < .001), assuming parallel prepolicy trends, and by 4.1 percentage points (95% CI, 2.1-6.1 percentage points; *P* < .001), assuming that differences in prepolicy trends continued after implementation of the policy (Table 2 and Figure 3). We observed a similar trend among reversible methods. Before the policy change, only 0.5% of Emergency Medicaid recipients (63 women) received a LARC method postpartum. The policy change was associated with an increase in LARC use by 18.7 percentage points (95% CI, 16.5-20.9 percentage points; *P* < .001), assuming parallel prepolicy trends, and by 17.7 percentage points (95% CI, 15.5-19.9 percentage points; *P* < .001), assuming differences in prepolicy trends would have continued after the policy. Similarly, before the policy, only 1.2% of Emergency Medicaid recipients (145 women) used a short-acting hormonal contraceptive method. The policy change was associated with an increase in use of short-acting hormonal methods by 7.8 percentage points (95% CI, 5.6-10.0 percentage points; *P* < .001), assuming parallel prepolicy trends, and by 6.4 percentage points (95% CI, 4.2-8.6 percentage points; *P* < .001), assuming differences in prepolicy trends continued after the policy.

## Discussion

The findings of this cohort study contribute to a growing body of evidence demonstrating the multigenerational health complications and inequities associated with inadequate access to postpartum care and family planning.^20,21,22,23^ Using data incorporating information from vital statistics and Medicaid claims from 2 states, we found that expanding postpartum coverage for low-income immigrant women was associated with significantly improved attendance at a postpartum visit and significantly increased use of all types of contraceptive methods postpartum. When postpartum care was covered for women who would have qualified for Medicaid, except for their citizenship status, their rates of attendance at a postpartum visit and use of postpartum contraception increased to levels observed in the traditional Medicaid population.^23^

Importantly, we observed significant increases across different types of effective postpartum contraception, including tubal sterilization, LARC, and short-acting hormonal methods. This finding indicates that women had access to the full range of contraceptive methods. Postpartum contraception is a critical component of comprehensive postpartum care because the ability to decide whether or when to become pregnant again has significant health consequences for the woman, her infant, and any future pregnancies.^1,24,25,26^

The Emergency Medicaid population is predominantly Latina and experiences increased rates of gestational and preexisting diabetes.^14^ Postpartum care for women with diabetes is a critical time to maximize glycemic control to promote long-term maternal health and prevent any future pregnancy complications (eg, fetal anomalies, macrosomia, or large for gestational age) should she choose to become pregnant again.^5,27^ Previous studies have found that postpartum contraception is protective against preterm birth, with every month of coverage decreasing the risk of a preterm birth by 1.1% (odds ratio, 0.98; 95% CI, 0.98-0.99).^3^

Providing all individuals who are low-income, regardless of citizenship, the choice of postpartum contraception is both an important opportunity to promote reproductive justice and maternal health and a potential cost-saving strategy for Medicaid.^13,28,29,30^ Currently, only a handful of states explicitly cover postpartum care for people who are low-income and noncitizens. Although federal policy changes have allowed for the option to expand prenatal care to low-income immigrants using federal funds, states must cover the entirety of postpartum care for this population. Few states have chosen to cover postpartum care for undocumented or recent immigrants, thereby propagating the maternal health crisis and contributing to generational inequity.^17,19^ Maternal health advocates have worked hard to expand Traditional Medicaid’s postpartum benefits to 1 year following childbirth, recognizing the importance of this period for maternal and child health. To date, Emergency Medicaid has been excluded from this dialogue.

### Limitations

Our study should be interpreted with the following limitations in mind. The use of administration data means that our data are subject to errors in coding. However, our study is strengthened by the use of 2 distinct data sources, Medicaid claims and birth certificate data, which allowed us to corroborate health outcomes and improve the demographic information available. We were unable to capture limited English proficiency, country of origin, and length of time in the US in our analyses. We used data from 2 states, Oregon and South Carolina, which may limit our generalizability to other areas. We were not able to capture subsequent births to women who moved out of state or switched to a private payor or who may have received care through charity programs, safety net clinics, or federally qualified health centers.

## Conclusions

In this cohort study, expanding postpartum coverage to the Emergency Medicaid population was associated with increased postpartum care attendance and contraceptive use. Postpartum care and contraception is a win-win policy solution: it improves health outcomes and reduces public costs.^28,29,30^ The Emergency Medicaid population should not be excluded from the national dialogue on postpartum Medicaid reform. The findings of this study suggest that significant gains may result from their inclusion.

## Notes

Supplement. **eTable 1.** List of Medical Billing Codes Used in the Study to Identify Postpartum Visit Services **eTable 2.** List of Medical Billing Codes Used in the Study to Identify Receipt of Postpartum Contraception **eTable 3.** Demographics and Standardized Mean Differences of Births by Race/Ethnicity, Emergency Medicaid, 2014-2019 **eTable 4.** Estimation of Differential Changes in Oregon Compared to South Carolina From Expansion of Postpartum Coverage, Full Models, 2014-2019 **eTable 5.** Differential Changes From the Prepolicy Period for Latinas Through 2019, Comparing Oregon to South Carolina, 2014-2019
