# Supplementary material for: Analysis of Contraceptive Use Among Immigrant Women Following Expansion of Medicaid Coverage for Postpartum Care
Source: JAMA Netw Open. 2021 Dec 15;4(12):e2138983. doi: 10.1001/jamanetworkopen.2021.38983 (PMC8674744; doi:10.1001/jamanetworkopen.2021.38983)
Supplement: Supplement. — eTable 1. List of Medical Billing Codes Used in the Study to Identify Postpartum Visit Services eTable 2. List of Medical Billing Codes Used in the Study to Identify Receipt of Postpartum Contraception eTable 3. Demographics and Standardized Mean Differences of Births by Race/Ethnicity, Emergency Medicaid, 2014-2019 eTable 4. Estimation of Differential Changes in Oregon Compared to South Carolina From Expansion of Postpartum Coverage, Full Models, 2014-2019 eTable 5. Differential Changes From the Prepolicy Period for Latinas Through 2019, Comparing Oregon to South Carolina, 2014-2019 [file jamanetwopen-e2138983-s001.pdf]

Supplemental Online Content

Rodriguez MI, Skye M, Lindner S, et al. Analysis of contraceptive use among immigrant women following expansion of Medicaid coverage for postpartum care. *JAMA Netw Open*. 2021;4(12):e2138983. doi:10.1001/jamanetworkopen.2021.38983

eTable 1. List of Medical Billing Codes Used in the Study to Identify Postpartum Visit Services
eTable 2. List of Medical Billing Codes Used in the Study to Identify Receipt of Postpartum Contraception
eTable 3. Demographics and Standardized Mean Differences of Births by Race/Ethnicity, Emergency Medicaid, 2014-2019
eTable 4. Estimation of Differential Changes in Oregon Compared to South Carolina From Expansion of Postpartum Coverage, Full Models, 2014-2019
eTable 5. Differential Changes From the Prepolicy Period for Latinas Through 2019, Comparing Oregon to South Carolina, 2014-2019

This supplemental material has been provided by the authors to give readers additional information about their work.

© 2021 Rodriguez MI et al. *JAMA Network Open.*

eTable 1. List of Medical Billing Codes Used in the Study to Identify Postpartum Visit Services

Code	Code Description
59400	Routine obstetric care including antepartum care, vaginal delivery (with or without episiotomy and/or forceps) and postpartum care
59410	Vaginal delivery only (with or without episiotomy and/or forceps); including postpartum care
59510	Routine obstetric care including antepartum care, cesarean delivery, and postpartum care
59515	Cesarean delivery only; including postpartum care
59610	Routine obstetric care including antepartum care, vaginal delivery (with or without episiotomy, and/or forceps) and postpartum care, after previous cesarean delivery
59614	Vaginal delivery only, after previous cesarean delivery (with or without episiotomy and/or forceps); including postpartum care
59618	Routine obstetric care including antepartum care, cesarean delivery, and postpartum care, following attempted vaginal delivery after previous cesarean delivery
59622	Cesarean delivery only, following attempted vaginal delivery after previous cesarean delivery; including postpartum care
57170	Diaphragm or cervical cap fitting with instructions
58300	Insertion of intrauterine device (IUD)
59430	Postpartum care only
99501	Home visit for postnatal assessment and follow-up care
0503F	Postpartum care visit
G0101	Cervical or vaginal cancer screening; pelvic and clinical breast examination
Z01.411	Encounter for gynecological examination (general) (routine) with abnormal findings
Z01.419	Encounter for gynecological examination (general) (routine) without abnormal findings
Z01.42	Encounter for cervical smear to confirm findings of recent normal smear following initial abnormal smear
Z30.430	Encounter for insertion of intrauterine contraceptive device
Z39.1	Encounter for care and examination of lactating mother
Z39.2	Encounter for routine postpartum follow-up
V24.1	Postpartum care and examination of lactating mother
V24.2	Routine postpartum follow-up
V25.11	Encounter for insertion of intrauterine contraceptive device
V25.12	Encounter for removal of intrauterine contraceptive device
V25.13	Encounter for removal and reinsertion of intrauterine contraceptive device

© 2021 Rodriguez MI et al. *JAMA Network Open.*

V72.31	Routine gynecological examination
V72.32	Encounter for Papanicolaou cervical smear to confirm findings of recent normal smear following initial abnormal smear
V76.2	Screening for malignant neoplasms of cervix
89.26	Gynecological examination

© 2021 Rodriguez MI et al. *JAMA Network Open.*

eTable 2: List of Medical Billing Codes Used in the Study to Identify Receipt of Postpartum Contraception

**Contraception Measure**	**Coding System**	**Codes**
Sterilization	ICD-9/ICD-10/CPT	0U574ZZ, 0U578ZZ, 0UL74CZ, 0UL74DZ, 0UL74ZZ, 0UL78DZ, 0UL78ZZ, 58340, 58565, 58600, 58605, 58611, 58615, 58670, 58671, 59510, 59618, 662, 6621, 6622, 6629, 74740, A4264, V252, V2651, Z302, Z9851
Implant	ICD-9/CPT	9963, 11981, A4260, J7306, J7307, S0180, V255, V4552
IUD	ICD-9/ICD-10/CPT	0UH97HZ, 0UH98HZ, 0UHC7HZ, 0UHC8HZ, 58300, 697, 99665, J7297, J7298, J7300, J7301, J7306, S4989, Q0090, S4981, T8331XA, T8332XA, T8339XA, T8359XA, T836XXA, V2511, V4551, Z975, Z30014, Z30430, Z30431
Oral Contraception	NDC	00008005601, 00008005602, 00008006201, 00008006202, 00008007501, 00008007502, 00008007801, 00008007802, 00008091202, 00008111720, 00008111730, 00008251101, 00008251102, 00008251401, 00008251402, 00008251403, 00008253301, 00008253302, 00008253303, 00008253501, 00008253505, 00008253601, 00008253603, 00008253605, 00008257601, 00008257602, 00009074630, 00009074631, 00009074634, 00009074635, 00009348404, 00009348405, 00009348406, 00009348410, 00009470901, 00009470913, 00009737601, 00009737602, 00009737603, 00009737604, 00009737607, 00009737611, 00023586228, 00023586230, 00025007107, 00025007124, 00025008109, 00025008124, 00025008184, 00025015107, 00025015124, 00025016109, 00025016124, 00025016184, 00025025203, 00025025403, 00025025706, 00025025906, 00025025912, 00025026306, 00025026506, 00025027206, 00025027212, 00025027406, 00025027412, 00047092635, 00047092735, 00047093011, 00047094211, 00047094435, 00047094735, 00052026106, 00052026108, 00052026906, 00052028106, 00052028306, 00052028308, 00062125100, 00062125101, 00062125115, 00062125120, 00062133115, 00062133215, 00062133220, 00062141101, 00062141116, 00062141123, 00062171215, 00062171400, 00062171415, 00062171420, 00062176015, 00062176100, 00062176115, 00062176120, 00062177015, 00062177115, 00062178015, 00062178022, 00062178100, 00062178115, 00062178120, 00062178122, 00062179515, 00062179600, 00062179615, 00062190015, 00062190115, 00062190120, 00062190215, 00062190315, 00062190320, 00062190700, 00062190715, 00062191000, 00062191015, 00062192001, 00062192015, 00071091315, 00071091345, 00071091347, 00071091348, 00071091547, 00071091548, 00071091647, 00071091648, 00071091715, 00071091745, 00071091747, 00071091748, 00071092815, 00071092847, 00087057841, 00087057941, 00087058342,

© 2021 Rodriguez MI et al. *JAMA Network Open.*

		00093214028, 00093214062, 00093313482, 00093313491, 00093330416, 00093330428, 00093330516, 00093330528, 00093531528, 00093531581, 00093531628, 00093531681, 00093532828, 00093532862, 00093542328, 00093542358, 00093542362, 00093566128, 00093566158, 00093603182, 00093603191, 00093614882, 00093614891, 00093807316, 00093807328, 00247052028, 00247059101, 00247069028, 00247069128, 00247069228, 00247100328, 00247100421, 00247139828, 00247151328, 00247151628, 00247151728, 00247176404, 00247176421, 00247176521, 00247198621, 00247198628, 00247200828, 00247201004, 00247201008, 00247201028, 00247201228, 00247201328, 00247214728, 00247216928, 00247217028, 00247223028, 00247223528, 00247226028, 00247226828, 00339651899, 00378334016, 00378334053, 00378655053, 00378655085, 00378727253, 00378727285, 00378727753, 00378728053, 00378728153, 378728253, 00378728285, 00378728353, 00378728485, 00378728490, 00378728590, 00378728753, 00378729253, 00378729653, 00378729753, 00378729785, 00378729853, 00378730053, 00378730153, 00378730185, 00378730653, 00378730685, 00378730753, 00378730785, 00378730853, 00378730885, 00378731553, 00378731585, 00378731685, 00430000531, 00430001005, 00430042014, 00430048214, 00430053014, 00430053514, 00430053550, 00430053595, 00430053714, 00430053750, 00430054014, 00430054050, 00430057014, 00430057045, 00430058014, 00430058045, 00430058114, 00430058214, 00430058311, 00430058514, 00430058545, 00536405548, 00536405648, 00536405748, 00536405844, 00536405944, 00555034458 00555071558, 00555900858, 00555900867, 00555900879, 00555900942, 00555900957, 00555900980, 00555901058, 00555901079, 00555901258, 00555901279, 00555901458, 00555901467, 00555901479, 00555901658, 00555901858, 00555902058, 00555902079, 00555902542, 00555902557, 00555902658, 00555902742, 00555902757, 00555902858, 00555903270, 00555903458, 00555903479, 00555904358, 00555904379, 00555904558, 00555904579, 00555904758, 00555904958, 00555904979, 00555905058, 00555905079, 00555905158, 00555905167, 00555905179, 00555906458, 00555906467, 00555906479, 00555906558, 00555906658, 00555906667, 00555906679, 00555912366, 00555913167, 00555913179, 00603359001, 00603359017, 00603359049, 00603751201, 00603751217, 00603751249, 00603752101, 00603752117, 00603752149, 00603752501, 00603752517, 00603752549, 00603754001, 00603754017, 00603754049, 00603760602, 00603760615, 00603760648, 00603760702, 00603760715, 00603760748, 00603760801, 00603760817, 00603760901, 00603760917, 00603761001, 00603761017, 00603761049, 00603762501, 00603762517,

© 2021 Rodriguez MI et al. *JAMA Network Open.*

		00603762549, 00603763401, 00603763417, 00603763449, 00603764017, 00603764201, 00603764217, 00603766301, 00603766317, 00603766517, 00703680101, 00703680104, 00703681121, 00781405815, 00781406015, 00781406215, 00781406615, 00781407515, 00781407552, 00781410315, 00781410352, 00781557515, 00781558307, 00781558315, 00781558336, 00781558436, 00781558491, 00781565615, 00781565815, 00905027928, 00905029128, 16714007301, 16714007304, 16714034001, 16714034004, 16714034601, 16714034604, 16714034701, 16714034704, 16714034801, 16714034804, 16714035901, 16714035903, 16714035904, 16714036001, 16714036004, 16714036301, 16714036304, 16714036501, 16714036504, 16714036603, 16714036701, 16714036704, 16714037001, 16714037003, 16714040401, 16714040404, 16714040501, 16714040504, 16714040601, 16714040604, 16714040701, 16714040703, 16714040801, 16714040803, 16714041301, 16714041304, 16714041601, 16714041603, 16714044001, 16714044004, 16714044101, 16714044104, 16714046401, 16714046404, 17314423101, 17314423102, 17478026006, 17478026028, 17478026106, 17478026128, 21695028128, 21695040701, 21695040703, 21695051428, 21695068528, 21695076901, 21695076928, 21695077001, 21695077028, 21695085501, 21695085601, 21695085603, 21695085701, 21695085703, 21695099528, 23490765301, 23490767001, 23490769901, 24090080184, 24090096184, 35356001468, 35356001568, 35356002168, 35356025528, 35356036128, 35356036328, 35356036528, 35356036828, 35356037028, 35356041128, 35356047605, 35356047628, 42254024228, 42254026028, 42254027028, 42254028703, 42254028728, 42987010023, 42987010124, 42987010719, 42987010813, 42987010923, 42987011014, 42987011061, 42987011124, 42987011128, 42987011423, 42987011427, 42987011524, 42987011528, 50102010000, 50102010001, 50102010003, 50102010010, 50102010048, 50102012001, 50102012003, 50102012010, 50102012048, 50102012801, 50102012803, 50102013001, 50102013003, 50102013010, 50102013048, 50102013090, 50102015401, 50102015403, 50102023311, 50102023313, 50102023511, 50102023513, 50419040201, 50419040203, 50419040301, 50419040303, 50419040501, 50419040503, 50419040603, 50419040701, 50419040703, 50419040803, 50419040872, 50419040901, 50419040903, 50419041021, 50419041112, 50419041128, 50419042408, 50419043203 50419043206, 50419043303, 50419043306, 50419043312, 50452025115, 50458017100, 50458017115, 50458017600, 50458017615, 50458017800, 50458017815, 50458017820, 50458019112, 50458019115, 50458019120, 50458019400, 50458019406, 50458019411, 50458019412, 50458019416, 50458019423, 50458019428, 50458019601,

© 2021 Rodriguez MI et al. *JAMA Network Open.*

		50458019615, 50458019712, 50458019715, 50458025100, 50458025106, 50458025115, 50458025128, 51285001728, 51285005866, 51285007997, 51285008070, 51285008198, 51285008297, 51285008370, 51285008498, 51285008787, 51285009158, 51285009287, 51285011458, 51285012058, 51285012570, 51285012698, 51285012797, 51285012870, 51285012998, 51285013197, 51285043165, 51285043187, 51285051428, 51285054628, 51285057628, 51660012786, 51660057286, 51862000701, 51862000706, 51862001201, 51862001206, 51862002801, 51862002806, 51862003601, 51862003603, 51862004501, 51862004591, 51862004701, 51862004791, 51862007201, 51862007206, 51862009701, 51862009706, 51862010001, 51862010006, 51862010201, 51862010206, 51862023801, 51862023803, 51862026001, 51862026006, 51862027901, 51862027906, 51862028401, 51862028403, 51862029201, 51862029206, 51862031801, 51862031803, 51862047001, 51862047006, 51862047101, 51862047106, 51862048965, 51862051001, 51862051006, 51862054501, 51862054506, 51862056401, 51862056406, 52544005431, 52544005841, 52544005872, 52544006431, 52544008728, 52544008741, 52544014328, 52544014331, 52544016528, 52544016541, 52544016731, 52544016741, 52544017572, 52544020431, 52544021028, 52544021928, 52544022829, 52544022891, 52544023328, 52544023341, 52544023528, 52544023531, 52544024531, 52544024728, 52544024828, 52544024928, 52544024941, 52544025428, 52544025928, 52544025931, 52544025988, 52544026528, 52544026531, 52544026829, 52544026884, 52544027428, 52544027431, 52544027621, 52544027721, 52544027928, 52544029021, 52544029128, 52544029231, 52544029241, 52544029528, 52544029531, 52544029831, 52544029841, 52544038328, 52544038428, 52544050721, 52544050821, 52544051021, 52544052628, 52544053221, 52544053321, 52544055028, 52544055228, 52544055321, 52544055428, 52544055628, 52544062928, 52544063028, 52544063128, 52544084721, 52544084728, 52544084828, 52544089228, 52544093528, 52544093628, 52544094028, 52544094928, 52544095021, 52544095121, 52544095328, 52544095428, 52544095928, 52544095931, 52544096691, 52544096728, 52544098128, 52544098131, 52544098228, 52544098231, 52959046004, 53002149001, 53002149003, 53002149006, 53002149101, 53002149103, 53002149106, 53002157701, 53002157703, 54569047300, 54569067200, 54569067300, 54569067400, 54569067800, 54569067900, 54569067902, 54569068500, 54569068501, 54569068900, 54569068901, 54569068902, 54569069000, 54569069500, 54569069501, 54569143900, 54569176600, 54569178003, 54569239700, 54569239701, 54569239900, 54569262300, 54569325401, 54569370100, 54569384400, 54569418400,

© 2021 Rodriguez MI et al. *JAMA Network Open.*

		54569420700, 54569422200, 54569422201, 54569426300, 54569426900, 54569427300, 54569427301, 54569471000, 54569481700, 54569487800, 54569487801, 54569489000, 54569490400, 54569498400, 54569499700, 54569499800, 54569499900, 54569511500, 54569516100, 54569527200, 54569534900, 54569535800, 54569543300, 54569549300, 54569549302, 54569552700, 54569555100, 54569579600, 54569579700, 54569579800, 54569581600, 54569582600 54569603200, 54569612800, 54569614400, 54569621900, 54569627200, 54569628000, 54569628100, 54569644800, 54569645500, 54569645600, 54569645700, 54569645800, 54569645900, 54569662000, 54569662200, 54569662300, 54569662400, 54868040400, 54868042800, 54868044200, 54868044300, 54868050200, 54868050700, 54868050800, 54868050801, 54868050901, 54868051600, 54868051800, 54868051801, 54868052500, 54868052801, 54868052901, 54868127600, 54868151200, 54868156400, 54868231600, 54868260600, 54868270100, 54868332800, 54868361300, 54868377200, 54868379000, 54868386300, 54868394800, 54868395100, 54868404500, 54868409300, 54868410000, 54868410001, 54868423900, 54868424000, 54868436800, 54868436900, 54868453800, 54868459000, 54868460700, 54868471200, 54868473000, 54868473100, 54868474200, 54868474400, 54868474500, 54868475400, 54868477600, 54868477800, 54868481400, 54868482800, 54868485000, 54868485100, 54868486000, 54868491100, 54868502800, 54868503100, 54868525700, 54868528600, 54868532600, 54868535600, 54868582600, 54868582800, 54868592200, 54868593500, 54868594200, 54868604400, 54868610000, 54868616100, 54868616200, 54868618300, 54868621000, 54868621300, 54868627200, 54868627300, 54868627400, 54868627500, 54868627600, 55045348506, 55045349701, 55045349801, 55045350501, 55045378106, 55045378206, 55289024504, 55289024608, 55289024708, 55289025308, 55289088704, 55887005228, 55887028628, 58016474701, 58016482701, 59762453701, 59762453702, 59762453801, 59762453802, 59762453809, 63187074828, 63187075428, 63187088928, 63187089028, 63187091128, 63629266601, 63955001001, 65162031658, 65162031684, 65162034784, 66993061128, 66993061528, 68180083711, 68180083713, 68180083811, 68180083813, 68180084011, 68180084013, 68180084311, 68180084313, 68180084411, 68180084413, 68180084611, 68180084613, 68180084811, 68180084813, 68180085411, 68180085413, 68180085711, 68180085713, 68180086011, 68180086012, 68180086411, 68180086413, 68180086511, 68180086513, 68180086611, 68180086613, 68180087311, 68180087313, 68180087511, 68180087513, 68180087611, 68180087613, 68180087711, 68180087713, 68180088011, 68180088013,

© 2021 Rodriguez MI et al. *JAMA Network Open.*

		68180088211, 68180088213, 68180088611, 68180088613, 68180089211, 68180089213, 68180089311, 68180089313, 68180089713, 68180089811, 68180089813, 68180089911, 68180089913, 68180090211, 68180090213, 68180090311, 68180090313, 68180090411, 68180090413, 68180091111, 68180091113, 68462013279, 68462013281, 68462030329, 68462030529, 68462030929, 68462030984, 68462031629, 68462031829, 68462031884, 68462038829, 68462038884, 68462039429, 68462039484, 68462055629, 68462055684, 68462056529, 68462063729, 68462063784, 68462064691, 68462064693, 68462067291, 68462067295, 68462071929, 68462071984, 68462072029, 68462072084, 68462073329, 68462073384, 69238103106, 69238103107, 69238153106, 69238155106, 69238155406, 69238158306, 69238160706, 75834011529, 75834011584, 75834011629, 75834011684, 76388028301
Patch	NDC	62192024, 50458019201, 50458019215, 50458019224, 54569541300, 54868467000
Ring	NDC	00052027301, 00052027303, 35356041003, 54569586500, 54868483200, 54868483201

© 2021 Rodriguez MI et al. *JAMA Network Open.*

eTable 3. Demographics and Standardized Mean Differences of Births by Race/Ethnicity, Emergency Medicaid, 2014-2019

Characteristic	No. (%)[a]		Standardized Difference
	All Other Race/Ethnicities[b] (n = 21 908)	Other/Unknown[c] (n = 5 759)	
State			1.93
Oregon	15295 (69.8)	170 (3.0)	
South Carolina	6613 (30.2)	5589 (97.0)	
Maternal age at birth, years			0.32
<20	673 (3.1)	499 (8.7)	
20-34	16012 (73.1)	4437 (77.0)	
≥35	5223 (23.8)	823 (14.3)	
Multiparous	17731 (80.9)	3558 (61.8)	0.43
County of residence			0.01
Metropolitan	16361 (74.7)	4147 (72.0)	
Non-Metropolitan	3385 (15.5)	1158 (20.1)	
Missing	2162 (9.9)	454 (7.9)	
Adequate prenatal care	18656 (86.4)	4209 (73.4)	0.33
Pregnancy complications	4381 (20.0)	746 (13.0)	0.19
Preterm birth	1701 (7.8)	464 (8.1)	0.01
Cesarean delivery	6006 (27.4)	1618 (28.1)	0.02

[a] Individual variable denominators differ depending on missingness.
[b] Category includes the subgroups “White,” “Black,” “Hispanic,” “Latina,” “Asian Indian,” “Chinese,” “Filipino,” “Japanese,” “Korean,” “Vietnamese,” “Other Asian,” “American Indian/Alaska Native,” “Native Hawaiian,” “Gaumanaian or Chamorro,” “Samoan,” and “Other Pacific Islander.”
[c] Category includes the subgroups “other,” and “unknown.”

© 2021 Rodriguez MI et al. *JAMA Network Open.*

eTable 4. Estimation of Differential Changes in Oregon Compared to South Carolina From Expansion of Postpartum Coverage, Full Models, 2014-2019

	Postpartum visit attendance		Receipt of Postpartum Contraception		Receipt of Postpartum Sterilization		Receipt of Postpartum LARC		Receipt of Postpartum Tier 2 Method	
	Assuming parallel trends	Assuming differential trends	Assuming parallel trends	Assuming differential trends	Assuming parallel trends	Assuming differential trends	Assuming parallel trends	Assuming differential trends	Assuming parallel trends	Assuming differential trends
Constant	0.043 (0.018 - 0.069)	-1.315 (-1.348 - -1.282)	-0.024 (-0.06 - 0.012)	1.779 (1.742 - 1.816)	-0.081 (-0.121 - -0.041)	1.299 (1.256 - 1.342)	0.036 (0.021 - 0.050)	0.202 (0.186 - 0.217)	0.021 (0.012 - 0.030)	0.279 (0.269 - 0.288)
Maternal age at birth, years	<0.001 (-0.001 - <0.001)	<0.001 (-0.001 - 0.001)	<0.001 (-0.001 - 0.001)	<0.001 (-0.001 - 0.001)	0.001 (0.001 - 0.002)	0.001 (0.001 - 0.002)	-0.001 (-0.002 - -0.001)	-0.001 (-0.002 - -0.001)	<0.001 (<0.001 - <0.001)	<0.001 (-0.001 - 0.001)
County of residence (Ref: Metropolitan)										
Non-Metropolitan	0.076 (0.02 - 0.132)	0.03 (-0.025 - 0.084)	-0.029 (-0.068 - 0.01)	0.004 (-0.034 - 0.043)	-0.029 (-0.062 - 0.004)	-0.011 (-0.048 - 0.027)	0.003 (-0.007 - 0.014)	0.009 (-0.001 - 0.020)	-0.004 (-0.01 - 0.003)	0.005 (-0.003 - 0.014)
Missing	0.097 (0.054 - 0.140)	0.05 (0.002 - 0.098)	-0.014 (-0.041 - 0.012)	0.021 (-0.006 - 0.047)	-0.013 (-0.034 - 0.009)	0.007 (-0.014 - 0.028)	0.006 (-0.004 - 0.016)	0.012 (0.003 - 0.021)	-0.008 (-0.014 - -0.001)	0.002 (-0.007 - 0.010)
Cesarean delivery	0.004 (-0.008 - 0.016)	0.004 (-0.009 - 0.017)	0.15 (0.087 - 0.213)	0.149 (0.085 - 0.214)	0.164 (0.097 - 0.232)	0.164 (0.095 - 0.233)	-0.012 (-0.02 - -0.004)	-0.012 (-0.021 - -0.003)	-0.003 (-0.007 - 0.002)	-0.003 (-0.007 - 0.002)
Preterm birth	0.008 (-0.012 - 0.029)	0.009 (-0.013 - 0.032)	-0.009 (-0.03 - 0.012)	-0.01 (-0.029 - 0.008)	-0.008 (-0.027 - 0.01)	-0.009 (-0.027 - 0.009)	-0.002 (-0.007 - 0.002)	-0.002 (-0.007 - 0.003)	0.001 (-0.003 - 0.006)	0.001 (-0.004 - 0.006)
Year of delivery (Ref: 2014)										
2015	-0.048 (-0.075 - -0.020)	0.340 (0.313 - 0.366)	0.02 (-0.011 - 0.05)	-0.473 (-0.503 - -0.443)	0.021 (-0.006 - 0.048)	-0.35 (-0.378 - -0.323)	-0.002 (-0.007 - 0.004)	-0.049 (-0.055 - -0.044)	<0.001 (-0.006 - 0.006)	-0.073 (-0.08 - -0.066)
2016	0.001 (-0.04 - 0.042)	0.753 (0.711 - 0.795)	0.017 (-0.016 - 0.05)	-0.951 (-0.984 - -0.917)	0.021 (-0.007 - 0.05)	-0.711 (-0.741 - -0.681)	-0.001 (-0.006 - 0.004)	-0.093 (-0.098 - -0.088)	-0.004 (-0.008 - 0.001)	-0.146 (-0.152 - -0.141)

© 2021 Rodriguez MI et al. *JAMA Network Open.*

2017	-0.011 (-0.049 - 0.027)	1.104 (1.069 - 1.139)	0.021 (-0.01 - 0.052)	-1.42 (-1.449 - -1.391)	0.026 (<0.001 - 0.051)	-1.066 (-1.091 - -1.041)	-0.001 (-0.007 - 0.005)	-0.138 (-0.144 - -0.132)	-0.004 (-0.008 - <0.001)	-0.216 (-0.221 - -0.211)
2018	0.059 (0.013 - 0.106)	1.398 (1.347 - 1.449)	0.039 (-0.006 - 0.084)	-1.695 (-1.733 - -1.656)	0.017 (-0.018 - 0.051)	-1.298 (-1.333 - -1.264)	0.022 (0.011 - 0.033)	-0.142 (-0.153 - -0.132)	<0.001 (-0.009 - 0.009)	-0.254 (-0.264 - -0.244)
2019	0.094 (0.030 - 0.157)	1.707 (1.635 - 1.779)	0.044 (-0.021 - 0.109)	-2.046 (-2.104 - -1.989)	0.006 (-0.038 - 0.051)	-1.58 (-1.623 - -1.537)	0.039 (0.01 - 0.067)	-0.159 (-0.189 - -0.128)	-0.001 (-0.018 - 0.016)	-0.307 (-0.322 - -0.292)
Post-policy delivery	-0.026 (-0.061 - 0.009)	-0.088 (-0.135 - -0.042)	-0.021 (-0.05 - 0.008)	0.114 (0.090 - 0.138)	0.01 (-0.008 - 0.027)	0.087 (0.063 - 0.110)	-0.025 (-0.041 - -0.009)	0.010 (0.008 - 0.013)	-0.005 (-0.015 - 0.005)	0.017 (0.012 - 0.022)
Oregon residence	-0.033 (-0.081 - 0.014)	0.117 (0.074 - 0.159)	0.077 (0.053 - 0.101)	-0.231 (-0.259 - -0.202)	0.067 (0.044 - 0.091)	-0.156 (-0.174 - -0.139)	0.003 (<0.001 - 0.006)	-0.042 (-0.059 - -0.025)	0.006 (0.001 - 0.012)	-0.032 (-0.041 - -0.024)
Post-Policy delivery*Oregon residence	0.406 (0.341 - 0.471)	0.479 (0.413 - 0.546)	0.332 (0.311 - 0.354)	0.282 (0.258 - 0.306)	0.068 (0.048 - 0.088)	0.041 (0.02 - 0.063)	0.187 (0.164 - 0.209)	0.177 (0.156 - 0.198)	0.078 (0.058 - 0.097)	0.064 (0.042 - 0.085)

© 2021 Rodriguez MI et al. *JAMA Network Open.*

eTable 5. Differential Changes From the Prepolicy Period for Latinas Through 2019, Comparing Oregon to South Carolina, 2014-2019

				Difference-in-Difference Estimate (95% CI)[a]	
Measure	Pre-Policy Proportion in Oregon, No. (%) (n=9 976)	Post-Policy Proportion in Oregon, No. (%) (n=2 822)	Difference in Pre-Policy Trends, Oregon vs. South Carolina % (95% CI)[b]	Assuming Parallel Trends % (95% CI)	Assuming Differential Trends % (95% CI)
Postpartum visit within 60 days	848 (8.5)	1 616 (57.3)	-0.66 (-1.05 - -0.28)	41.3 (34.9 - 47.7)	48.2 (41.9 - 54.4)
Postpartum contraception within 60 days	906 (9.1)	1 288 (45.6)	0.42 (0.17 - 0.68)	35.1 (32.2 - 38.0)	30.9 (27.8 - 33.9)
Tier 1 - Sterilization	736 (7.4)	429 (15.2)	0.20 (-0.06 - 0.46)	6.4 (3.4 - 9.5)	4.5 (1.0 - 8.0)
Tier 1 - Interval LARC	55 (0.6)	602 (21.3)	0.10 (0.05 - 0.17)	20.4 (18.0 - 22.7)	19.3 (16.9 - 21.8)
Tier 2 - Hormonal methods	115 (1.2)	257 (9.1)	0.12 (0.05 - 0.19)	8.3 (6.2 - 10.3)	7.1 (4.9 - 9.2)

Abbreviation: LARC, Long-Acting Reversible Contraception

[a] Difference-in-difference estimates adjusted for maternal age, non-metropolitan location, cesarean delivery, and preterm gestational age. Standard errors clustered at the county level

[b] Pre-policy trend difference estimated from an interaction between a linear pre-policy time trend and Oregon residence, adjusted for maternal age, non-metropolitan location, cesarean delivery, and preterm gestational age. Standard errors clustered at the county level

[c] Assuming that differences between Oregon and South Carolina would have remained constant had the policy not been implemented in Oregon

[d] Assuming that the pre-policy differences between Oregon and South Carolina would have continued had the policy not been implemented in Oregon

© 2021 Rodriguez MI et al. *JAMA Network Open.*
